# The Role Descriptions of Triage Nurse in Emergency Department: A Delphi Study

**DOI:** 10.1155/2016/5269815

**Published:** 2016-06-13

**Authors:** Mohsen Ebrahimi, Amir Mirhaghi, Reza Mazlom, Abbas Heydari, Asra Nassehi, Mojtaba Jafari

**Affiliations:** ^1^Department of Emergency Medicine, Imam Reza Hospital, Mashhad University of Medical Sciences, Mashhad 9137913316, Iran; ^2^Evidence-Based Caring Research Center, Department of Medical-Surgical Nursing, School of Nursing and Midwifery, Mashhad University of Medical Sciences, Mashhad 9137913199, Iran; ^3^Research Center for Health Services Management, Institute for Futures Studies in Health, Kerman University of Medical Sciences, Kerman 7616913555, Iran; ^4^Department of Medical-Surgical Nursing, Faculty of Nursing and Midwifery, Bam University of Medical Sciences, Khalije Fars Boulevard, Bam, Kerman 7661771967, Iran

## Abstract

*Background*. Triage nurses play a pivotal role in the emergency department. However some researchers have attempted to expand triage nurse's role; remarkable discrepancies exist among scholarly communities. The aim was to develop a role description of triage nurse relying on the experts.* Methods*. A modified Delphi study consisting of 3 rounds was performed from March to October 2014. In the first round, an extensive review of the literature was conducted. Expert selection was conducted through a purposeful sample of 38 emergency medicine experts.* Results*. Response rates for the second and third rounds were 37% and 58%. Average age of panelists was (38.42 ± 5.94) years. Thirty-nine out of 54 items reached to the final round. Prioritizing had the higher agreement rate and least agreement on triage related interventions.* Conclusion*. Triage nursing as a relatively new role for nurses needs significant development to be practiced. Comprehensive educational programs and developmental research are required to support diagnostic and therapeutic interventions in triage practice by nurses.

## 1. Introduction

Triage is defined as prioritizing or sorting the patients for the care and treatment that is due to shortage of the necessary resources in the emergency department (ED) [1]. Several studies have focused on the validity and reliability of triage scales [2, 3] and assessed agreement between nurses and physicians [4, 5]. Factors which affect triage practice were also studied [6]. Some studies have explored triage nurse's daily job activities as well as how they decided to allocate patients to triage categories [7, 8] but researchers rarely try to describe triage nurse job.

In response to overcrowding, some studies tried to explain increased efficiency of triage process regarding expanding physician and nurses roles in the triage room [9–12] in which placing a senior emergency physician with the triage nurse reduced waiting times for nonemergent cases [10] so the concept of team triage has been evolved. Of course, it is clear that it is not possible for most EDs to have a senior emergency physician in the triage room. In order to customize patient flow more consciously and despite high satisfaction among staff and patient, initiating X-rays by triage nurses did not reduce transit times for patients with limb injuries in ED [13]. In contrast, some studies have indicated triage nurse ordering seems to be an effective intervention to reduce ED length of stay in patients with injuries [11]. Therefore findings revealed that triage nurses' role is challenging in the ED.

Role description is designed to guarantee the best possible performance by employees. In this way, the emergency associations have tried to develop position statement to define triage nurse role in order to secure patient safety in triage room. All associations have indicated that triage must be performed at least by a registered nurse. Despite remarkable discrepancies among statements, triage nurses are required to perform prioritizing of the patient care, take educational programs, and provide a safe environment as well as interpersonal qualifications needed to fulfill this role [14–17]. However, only the Emergency Nurses Association (ENA) has comprehensively indicated several educational programs (CPR, ALS, ENPC, TNCC, GENE, CEN, and CPEN) in addition to other triage educational programs [14]. Furthermore interpersonal qualifications including interpersonal, interdisciplinary, critical thinking, and communication skills as well as accurate decision making have been stated by several emergency associations [14–17]. However advanced practice nursing roles have been recommended to improve patient flow through the ED [18]; advanced practice nursing roles have been rarely addressed by emergency associations. Even the Royal College of Nursing has indicated triage related interventions such as administrating analgesia [17], so it concluded that role description of triage nurse suffered from lack of an integrated approach. There is a strong possibility that it also affects the reliability and validity of decisions among triage nurses in the ED.

Delphi method as an iterative process is to explore implicit information leading to differing judgments and search information which may generate a consensus on behalf of the respondent group [19]. It has been employed by many nurse researchers in a wide variety of studies [20]. So Delphi method was used to develop a role description of triage nurse relying on the emergency medicine experts.

## 2. Materials and Methods

A modified Delphi study consisting of 3 rounds was performed from March to October 2014. Anonymity, iteration with controlled feedback, and statistical consensus were included in the Delphi method [20].

The study was approved by the Research Ethics Committee at the Mashhad University of Medical Sciences. Confidentiality and anonymity of the experts' participation were secured. It was clearly defined that there is no obligation for them to reply to the questionnaire.

**Figure 1:**
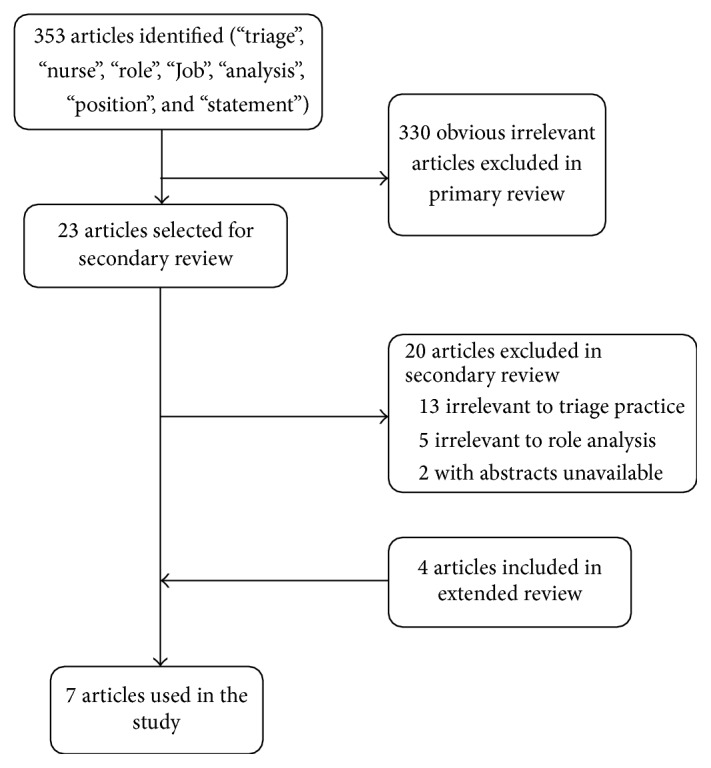
Study selection process.

In the first round, an extensive review of the literature was conducted [21]. Main question of the study was, what are the attributes of the triage nurse role? Electronic databases were searched from inception to February 2014. Medline (Pubmed) and Scopus were searched without any additional filter. The keywords searched alone and in combination were “triage”, “nurse”, “role”, “Job”, “analysis”, “position”, and “statement”. Titles and abstracts were reviewed to identify the relevant studies in the primary review. In the secondary review, articles were included only if the study defined role of the triage nurse. Studies were excluded if they have not been approved by emergency associations or colleges. Extended review was conducted to retrieve additional studies using search engines. Data were extracted from relevant articles based on researchers' agreement to identify attributes of the triage nurse contributing to the generation of items pool to compose the primary questionnaire. Researchers continued to collect data until they reach a point of data saturation. In the first round, selecting items for primary questionnaire were based on the three researchers' consensus. Each researcher rated relevancy of items using Likert-type scale items (5: completely, 4: mostly, 3: moderately, 2: slightly, and 1: not at all). Average of rated responses was reported for each item (Figure 1).

Expert selection was conducted through a purposeful sample of 38 emergency medicine experts and emergency nursing scholars. They were involved in emergency medicine and emergency nursing, so they were invited to take part. Experts were ED directors and physicians or nurses who have known interest in triage practice and research. Potential contributors were retrieved from triage committees and associations, authors of triage publications, and published articles. All contributors were individually communicated by email.

In the second round, an initial task list of triage nurse role was sent to the expert panelists in May 2014. Fifty-four items were included in the questionnaire. Items were related to the domains including basics, ethics, triage assessment, decision making, informing, competencies, environment, and documentation. Expert opinions on proposed tasks were obtained by responses on a 5-point Likert scale (1: strongly disagree, 2: disagree, 3: undecided, 4: agree, and 5: strongly agree). Participants were asked to comment on any items as needed. Completed questionnaires were analyzed and agreement on each item was reported.

In the third round, a modified questionnaire was sent to the expert panelists in July 2014. Participants were informed about the opinion of other colleagues as well as theirs. Both second- and third-round questionnaires were sent to all the individuals regardless of their contributions in the second round. Two reminders sent to the participants who did not reply. Reminders were sent at two-week interval.

Proportions and percentages were calculated for ordinal data of Likert-type response scale. The data of each iterative round was analyzed using MS Excel (2007). Only strongly agree and agree responses to items were assumed as approved items [22]. Each approved item which obtained a consensus level of 80% was identified for the next phase. The feedback for each round contained statistical result from the former round, which included the consensus level reached for each item.

## 3. Results

Six expert panelists of 38 were female. Average age of panelists was (38.42 ± 5.94). Most participants had a medical degree in emergency medicine. All participants had an affiliation with a university hospital.

### 3.1. Round One

Twenty-three studies were chosen among 353 articles which have been retrieved through searching databases and 7 were included in the final analysis. Sixty-two items were primarily extracted from the final studies and redundant items were merged together, remaining 54 items in the list (Table 1).

### 3.2. Round Two

Response rate for the second round was 31% (14/38). The number of items which remained in the end of the round was 43 (80%). Forty-one percent (22/54) of items achieved a complete consensus. 11 items were excluded since they did not reach a consensus level of 80%. Excluded items included legal issues (triage nurse responsibility for patient status and handing over level I patients to the emergency nurses and reconsidering their decisions); informing issues (informing patients about assigned triage level, their condition, outcomes, and potential care and treatment); ethical issues (empathy and tactfulness characteristics); and other issues (mandatory research participation and responsibility for triage room facilities and condition). Four new items were proposed including diagnostic and therapeutic interventions and added to the next round questionnaire (49 items). Four (7%) items were suggested to be edited lightly by participants (Table 1).

### 3.3. Round Three

Response rate for third round was 58% (22/38). The number of items which reached to the end of the round was 43 (88%). Fifty-five percent (27/49) of items achieved a complete consensus. Seven items were excluded since they did not reach a consensus level of 80%. Excluded items included ethical issues (having patience); competency issues (continuing education); and other issues (diagnostic and therapeutic intervention and physician participations). No item was proposed for the next round questionnaire (39 items) (Table 1).

## 4. Discussion

Triage related interventions still need extensive development to be reliable enough to practice by triage nurses. Our results did not reveal substantial agreement on triage nurse ordering (Table 1) which is consistent with most position statements on triage nurse role [14–17]. However some studies supported diagnostic interventions by triage nurse as well as advanced practice nursing roles [11, 18, 23]; scientific evidence is limited to ensure significant impact on patient flow in the emergency department and efficiency [24]. Despite this inconsistency, expanding the role of triage nurse caused significant increases in nurses' satisfaction [12]. Further studies are needed to develop more clarification of the contextual factors related to the interventions and investigate the impact of them on the ED measures as the next generation of triage studies. Educational preparation and organized teamwork are essentially required for advanced practice role to be successfully implemented [25]. So prioritizing care and introducing fast track for patients were known as the fundamental role for triage nurse which is unique to the emergency department and associated with desirable outcomes [24].

There is broad consensus that nurses are accountable for ensuring safe triage practices [14–17]. Our results did not indicate a broad consensus among clinicians that physicians routinely perform triage in ED (Table 1). Several studies demonstrated that intelligent use of physicians in triage causes substantial improvement in ED patient flow and results in shorter length of stay especially in overcrowded conditions in the ED [24, 26]. Some studies suggested that emergency nurse practitioners as an optimal choice have greater impact on quality measures and financial indices in ED [12, 27]. Comparison between the nurse practitioner and general practitioners demonstrated that the nurse practitioner can deal with patients effectively [12, 28] and they can be assumed as superb candidates for expanded and extended role [29].

A limitation of the study is that the modified Delphi method was used. Several modifications of the original Delphi method have been described in the literature [30] and standardized definitions of these modifications are not available. Besides, panelists did not generally mention rationale in case of disagreement.

## 5. Conclusions

Triage nursing as a relatively new role for nurses is a challenging role in a dynamic environment which needs significant development to be practiced. Prioritizing is defined as the pivotal role for triage nurse. Comprehensive educational programs and developmental research are required to support diagnostic and therapeutic interventions in triage practice by nurses.

## Figures and Tables

**Table 1:** Participants' responses to the questionnaire.

Items	First round% (*n* = 3)	Second round% (*n* = 14)	Third round% (*n* = 22)
Prioritizing patients must be based on patients' acuity	100	86	91
Prioritizing patients must not be affected by ED overcrowding and financial status of patients	66	86	100
Triage decisions must be made based on evidence	100	100	100
Triage decisions must be made based on reliable and valid algorithm	100	100	100
Triage nurse is responsible for patients status until the first physician visit	66	71^*∗*^	—
Patients must be assigned to the triage levels based on relevant acuity	100	100	100
Nurses are not allowed to reject patients from ED	100	93	95
Nurses must be committed to the patient bill of rights	100	100	100
Nurses must identify themselves to patients	100	100	100
Nurses must have empathy toward patients	66	64^*∗*^	—
Patients' culture and value must be respected by triage nurses	100	93	100
Triage nurses must manage ethical conflict in triage decisions	100	93	91
Triage nurses must manage aggressive patients effectively	100	93	86
Triage nurses must have patience	100	100	77^*∗*^
Triage nurses must handle conflict in a tactful manner	100	57^*∗*^	—
Triage nurses must have interpersonal skills	100	100	100
Triage nurses must perform the first assessment in the ED	100	100	100
Triage nurses must estimate life-threatening risk of chief complaints	100	100	100
Except in life-threatening conditions, triage nurses must observe patients at least in 2 minutes	66	86	82
Triage nurses must prioritize patients based on assessing respirations, pulse rate, blood pressure, temperature, O_2_ saturation, and other diagnostic measures	66	93	100
Patients should be reassessed when needed	100	100	100
Triage decisions must be recorded	100	100	100
Triage nurses must follow organization's guidelines during decision making	66	86	90
In case of doubt, triage nurses must consult with attending physician or head nurse	100	93	100
In case of doubt, triage nurses must assign patient to the higher level of acuity	100	100	100
Triage nurses must reconsider their decisions if requested from medical directors	100	64^*∗*^	—
Level I patients must be directed to the CPR room promptly	100	93	100
Level I patients must be handed over to the emergency nurses	100	71^*∗*^	—
Patients must be informed in either verbal or written way regarding their assigned triage level	66	64^*∗*^	—
Patients must be informed that they must inform triage nurses in case of deterioration in their health status	100	86	77^*∗*^
Triage nurses must explain waiting time to the first visit	66	93	82
Triage nurses must explain necessary information relating to the patient condition	100	71^*∗*^	—
Triage nurses must explain necessary information relating to alternative facilities for care and treatment	100	71^*∗*^	—
Triage nurses must be aware of alternative health care facilities	100	93	91
Triage nurses must have a minimum degree of BS in nursing	100	93	82
Triage nurses must have a minimum 2-year experience in ED	100	100	100
Triage nurses must have a minimum 40-hour educational courses annually	66	100	73^*∗*^
Nurses must be knowledgeable about clinical semiology	100	100	100
Nurses must be knowledgeable about advanced and basic adult life support	100	86	82
Nurses must be knowledgeable about emergencies	100	100	100
Nurses must be knowledgeable about gynecological, maternal, neonatal, children, and geriatrics emergencies	100	100	100
Nurses must be knowledgeable about outcome of disease	100	71^*∗*^	—
Nurses must participate in at least one emergency research	100	50^*∗*^	—
Triage room must be somewhere between emergency department and security room	100	100	100
Triage room must be easily recognizable for patients	100	100	100
Triage room must have an appropriate view on entrance of ED	100	100	100
Triage room must be accessible for ambulances, stretchers, and wheelchairs	100	100	100
Triage room must be at least 12 square meters	100	79^*∗*^	—
Triage room must be equipped for professionals standard infection control precautions	100	93	82
Triage room must have alarm, telephone, and closed-circuit television	100	100	100
Triage nurses are responsible for facilities of triage room	100	79^*∗*^	—
Triage nurses are not permitted to leave the triage room, except for handing over level I patients	100	86	77^*∗*^
Triage nurses must document triage assignments	100	93	100
Triage nurses must report daily and monthly statistics of triage	100	93	100
Triage nurses must consider hospital facilities when assigning patients to triage level	—	Proposed	82
Diagnostic related interventions must be performed by triage nurses	—	Proposed	41^*∗*^
Therapeutic related interventions must be performed by triage nurses	—	Proposed	55^*∗*^
Physician must participate in prioritizing of patients in triage room	—	Proposed	77^*∗*^

^*∗*^Less than consensus level of 80%.

## Appendix

### Search Strategies

*Primary Search*. Primary search included the following: (“triage” [MeSH Terms] OR “triage” [All Fields]) AND (“nurse's role” [MeSH Terms] OR (“nurse's” [All Fields] AND “role” [All Fields]) OR “nurse's role” [All Fields] OR (“nurse” [All Fields] AND “role” [All Fields]) OR “nurse role” [All Fields]).
